# Determinants of long‐term paramagnetic rim lesion evolution in people with multiple sclerosis

**DOI:** 10.1002/acn3.52253

**Published:** 2024-11-18

**Authors:** Jack A. Reeves, Alexander Bartnik, Maryam Mohebbi, Murali Ramanathan, Niels Bergsland, Dejan Jakimovski, Gregory E. Wilding, Fahad Salman, Ferdinand Schweser, Bianca Weinstock‐Guttman, David Hojnacki, Svetlana Eckert, Francesca Bagnato, Michael G. Dwyer, Robert Zivadinov

**Affiliations:** ^1^ Buffalo Neuroimaging Analysis Center, Department of Neurology, Jacobs School of Medicine and Biomedical Sciences University at Buffalo, State University of New York Buffalo New York USA; ^2^ Department of Pharmaceutical Sciences State University of New York Buffalo New York USA; ^3^ Department of Biostatistics, School of Public Health and Health Professions State University of New York at Buffalo Buffalo New York USA; ^4^ Center for Biomedical Imaging at the Clinical Translational Science Institute University at Buffalo, State University of New York Buffalo New York USA; ^5^ Jacobs Neurological Institute Buffalo New York USA; ^6^ Department of Neurology, Jacobs School of Medicine and Biomedical Sciences University at Buffalo, State University of New York Buffalo New York USA; ^7^ Neuroimaging Unit, Neuroimmunology Division, Department of Neurology Vanderbilt University Medical Center Nashville Tennessee USA; ^8^ Department of Neurology, Nashville VA Medical Center Tennessee Valley Healthcare System Nashville Tennessee USA

## Abstract

**Objective:**

Baseline paramagnetic rim lesion (PRL) load predicts disease progression in people with multiple sclerosis (pwMS). Understanding how PRLs relate to other known MS‐related factors, and the practical utility of PRLs in clinical trials, is crucial for informing clinical decision‐making and guiding development of novel disease‐modifying treatments (DMTs).

**Methods:**

This study included 152 pwMS enrolled in a larger prospective, longitudinal cohort study who had 3T MRI scans and clinical assessments at baseline and 5‐ or 10‐year follow‐ups. PRLs were identified on baseline 3T quantitative susceptibility maps and classified as persisting, disappearing, or newly appearing at follow‐up. The relationships between PRL evolution and clinical, radiological, environmental, and genetic characteristics were assessed, and clinical trial sample sizes were estimated using PRL appearance or disappearance as outcome measures.

**Results:**

DMT use was associated with lower odds of new PRL appearance (for high‐efficacy DMTs: odds ratio = 0.088, *p* = 0.024), but not disappearance. Current smoking status was associated with greater baseline PRL number (*B* = 0.527 additional PRLs, *p* = 0.013). A 24‐month clinical trial in people with progressive MS for a DMT that doubles the rate of PRL rim disappearance would require an estimated 118 people with progressive MS per group at 80% statistical power.

**Interpretation:**

Early MS diagnosis and subsequent DMT initiation may reduce new chronic active inflammation. However, the utility of PRL disappearance or new PRL appearance as outcome measures in clinical trials is limited by potentially large sample sizes that are needed for moderate efficacy drugs.

## Introduction

Chronic active lesions (CALs) are increasingly recognized as an indicator of multiple sclerosis (MS) disease progression.^1^ CALs exhibit substantial myelin and axonal damage within their core and ongoing axonal deterioration at their periphery, accompanied by iron‐rich microglia.^2^ These lesions can manifest as “paramagnetic rim lesions” (PRLs) on iron‐sensitive MRI due to the rim of iron‐rich microglia, or on PET scans and longitudinally on T1‐weighted and T2‐weighted images as “slowly expanding lesions”.^3^, ^4^ Compared to non‐PRL T2 lesions, PRLs tend to have stronger T1 hypointensity, indicative of greater axonal damage.^5^ There is some overlap between PRLs and slowly expanding lesions, with PRLs that persist over long periods of time tending to have greater volume increases than non‐PRL T2 lesions, but these two types of lesions are largely disjoint.^6^, ^7^ Recent studies have linked PRLs with a more pronounced disability progression, faster transition to progressive MS, and more frequent clinical relapses.^8^, ^9^, ^10^ These findings suggest the potential of PRLs as a valuable imaging marker of chronic inflammation in clinical trials targeting MS disease progression.

Previous studies have shown that approximately 18–40% of acute‐enhancing lesions convert to PRLs.^11^, ^12^ However, these studies had short follow‐up times (mean follow‐up times <15 months). PRLs can expand and cause continued tissue destruction over 7+ years,^13^ so studies with comparably long follow‐up times are needed for a complete picture of PRL evolution. Additionally, the overall incidence rate of new PRL appearance is unknown, due to a paucity of longitudinal studies with sufficient sample sizes and follow‐up times, hampering its translation as an endpoint for phase II and III clinical trials.

Recently, several genetic and environmental factors have been linked to disease severity in people with MS (pwMS). For example, several genetic variants associated with MS severity have been reported,^14^ including the HLA‐DRB1*1501 allele, which is associated with long‐term disability worsening and related to increased inflammatory activity.^15^ Lower vitamin D levels and smoking^16^, ^17^, ^18^ also negatively affect both clinical and imaging outcomes. Moreover, Epstein–Barr virus has been strongly implicated in having a causal role in the development of MS as well as being linked to more severe cerebral pathology in pwMS.^19^, ^20^ Nevertheless, it remains largely unknown as to how any of the aforementioned factors relate to PRLs. This is a key missing piece in understanding chronic active inflammation which could help guide the development of novel disease‐modifying treatments (DMTs).

In this study, we classified PRLs on baseline and follow‐up scans and assigned them as persisting, disappearing, or newly appearing at follow‐up. In addition to gadolinium (Gad)‐enhancing lesions, we evaluated previously unexplored clinical, radiological, genetic, and environmental associations of long‐term PRL appearance, disappearance, and volume change. Finally, we performed power analysis based on our observed rates of PRL appearance and rim disappearance to inform design of future clinical trials.

## Methods

### Study population

Subjects were part of a prospective, longitudinal cohort study that investigated cardiovascular, environmental, and genetic factors in MS (CEG‐MS).^21^ CEG‐MS included individuals who were: (1) aged 18–75 years, and (2) met the 2010 revision of the McDonald criteria for the diagnosis of MS or clinically isolated syndrome (CIS). CEG‐MS exclusion criteria included: (1) contraindications for MRI examination, (2) pregnancy or nursing, and (3) experiencing a clinical relapse or undergoing intravenous corticosteroid therapy within 30 days of the MRI examination. All people with CIS (diagnosed using the 2010 McDonald criteria) met the 2017 McDonald criteria for relapsing–remitting MS (RRMS) and were included in the RRMS group.^22^ The CEG‐MS study was approved by the Institutional Review Board of the University at Buffalo and all participants provided written informed consent in accordance with the Declaration of Helsinki. Additional inclusion criteria for the present study included availability of baseline and follow‐up MRI (including T2‐FLAIR, 3D T2*‐weighted gradient echo, and post‐contrast T1‐weighted (T1w) scans for PRL analysis), and a clinical assessment within 30 days of each scan. Subsets of pwMS had information on cigarette pack‐years at baseline and smoking status at baseline and follow‐up, baseline HLA DRB1*1501 genotype, or baseline serum concentrations of anti‐Epstein–Barr nuclear antigen 1 (EBNA‐1) titer or 1,25‐dihydroxyvitamin D3 (calcitriol). Participants were excluded from the current study if artifacts on their MRI scans prevented analysis.

### Genotype analysis of HLA DRB1*1501

HLA DRB1*1501 status was determined by genotyping DNA from peripheral blood for the rs3135005 single nucleotide polymorphism using allele discrimination (*Assays‐on‐Demand genotyping kit, Applied Biosystems, Redwood City, CA*) as previously described.^23^ Subjects were classified as having the AA allele (high risk for MS susceptibility), GA allele, or GG allele.

### Serum evaluation of anti‐EBNA‐1 IgG antibody and calcitriol

Serum anti‐EBNA‐1 IgG antibody levels were quantified with an enzyme‐linked immunosorbent assay (ELISA) kit (Diamedix Corporation, Miami, FL, USA) and normalized to the manufacturer's cut‐off standards as previously described.^20^ Serum anti‐EBNA‐1 titer levels were expressed as arbitrary units per mL and log‐transformed prior to analysis.

Vitamin D metabolites, including calcitriol, were quantified using liquid chromatography–tandem mass spectrometry as described elsewhere.^24^ Calcitriol serum concentrations were log‐transformed prior to analysis.

### MRI acquisition protocol

Imaging was performed on the same 3T scanner (Signa Excite HD 12.0; GE, Milwaukee, WI, USA) using an eight‐channel head‐and‐neck coil and using the same three‐dimensional (3D) gradient‐echo sequence with first‐order flow compensation in read and slice directions (matrix, 512 × 192 × 64; 0.5 × 1 × 2 mm^3^; 12° flip; echo time (TE) = 22 ms; repetition time (TR) = 40 ms; bandwidth, 13.89 kHz). The following additional sequences were acquired during the same imaging session for all subjects: spin‐echo T1w imaging (matrix, 256 mm ×192 mm; FOV, 256 mm × 192 mm; TE = 16 ms; TR = 600 ms); FLAIR (matrix, 256 mm × 192 mm; FOV, 256 mm × 192 mm; TE = 120 ms; inversion time (TI) = 2100 ms; TR = 8500 ms; flip angle = 90°; echo‐train length, 24); dual fast spin‐echo proton density‐ and T2‐weighted imaging (matrix, 256 mm × 192 mm; FOV, 256 mm × 192 mm; TE1 = 9 ms; TE2 = 98 ms; TR = 5300 ms; echo‐train length = 14); and a 3D high‐resolution T1w inversion recovery fast spoiled‐gradient echo (IR‐FSPGR) (TE = 2.8 ms; TI = 900 ms; TR = 5.9 ms; flip angle, 10°; isotropic 1 mm resolution). The spin‐echo T1w image was repeated after administration of gadolinium.

### Reconstruction of magnetic susceptibility maps (QSM)

GRE images were reconstructed from raw k‐space data (512 × 512 × 64 spatial matrix) with scalar phase matching for phase images and sum‐of‐squares for magnitude images.^25^ Before processing, K‐space data were zero‐padded in the phase‐encoding direction to achieve an isotropic in‐plane resolution. Imaging gradient non‐linearity distortions were corrected for as described previously.^26^ Phase images were unwrapped with the best‐path algorithm^27^ and then underwent background field removal (BFR) using RESHARP.^28^, ^29^ The HEIDI algorithm was applied to the background‐corrected field map to solve the ill‐conditioned QSM dipole inversion. The BFR and inversion algorithms were selected due to a recent study showcasing their high sensitivity and reproducibility.^30^, ^31^

### MRI analyses

**Figure 1:**
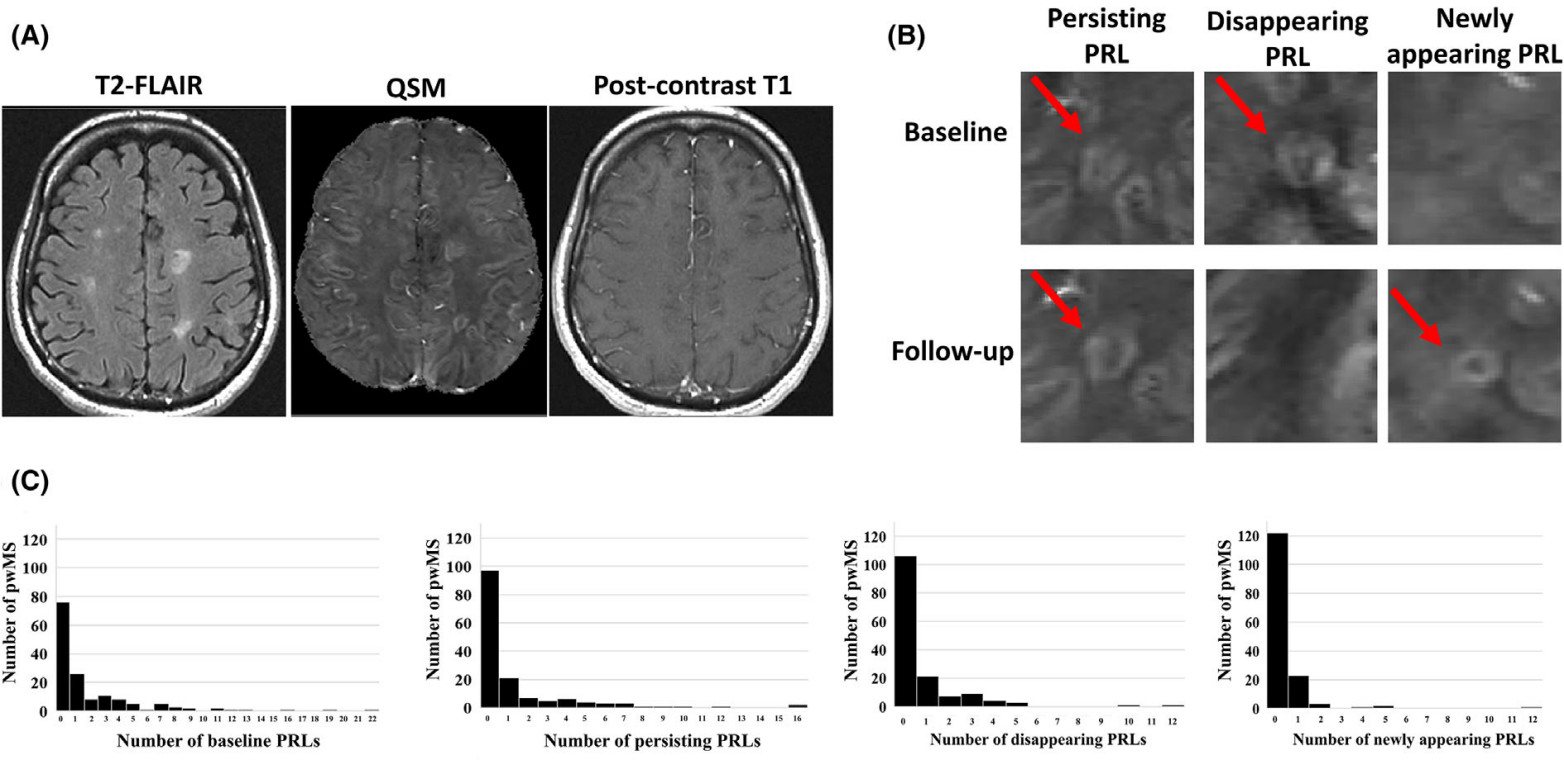
(A) Sample T2‐FLAIR, QSM, and post‐contrast T1 images. (B) Examples of persisting, disappearing, and newly appearing PRLs on QSM at baseline and follow‐up (intensity range −0.1 to 0.2). (C) Histogram distributions of number of baseline PRLs, persisting PRLs, disappearing PRLs, and newly appearing PRLs per pwMS.

T2‐, T1 black hole‐ (T1BH), and Gad‐enhancing lesion volumes (LV) were quantified using a semi‐automated contouring technique.^32^ The QSM reconstruction methodology has been thoroughly detailed elsewhere.^33^ A researcher with 3 years of lesion classification experience (J.R.) identified and semi‐automatically delineated PRL regions of interest on QSM images, while also referencing co‐registered T2‐FLAIR and T1w post‐contrast images. Subsequently, senior researchers with 10+ years of lesion classification experience analyzed the QSM, T2‐FLAIR, and T1w post‐contrast images for PRLs (N.B., M.D., and R.Z.). PRL classifications were deliberated in review meetings until a group consensus was achieved. Initial PRL classifications were performed on baseline and follow‐up images independently. Baseline and follow‐up QSMs from the same individuals were then linearly co‐registered; PRL ROIs were separated using the FSL “cluster” function and PRL classifications, indices, and volumes reconciled between baseline and follow‐up scans; and PRLs were assigned as persisting (rim present at baseline and follow‐up), newly appearing (rim present only at follow‐up), or disappearing (rim present at baseline but not follow‐up). Figure 1 shows sample T2‐FLAIR, QSM, and post‐contrast T1‐gad images, examples of persisting, newly appearing, and disappearing PRLs (with hyperintense (bright) rims on QSM), and histogram distributions of per‐pwMS numbers of PRL subtypes. Subsequently, each persisting PRL was evaluated for whether it spatially corresponded with a T1BH lesion at the baseline and/or follow‐up timepoint. Each disappearing PRL was evaluated for whether it corresponded with a T1BH lesion at the baseline timepoint, and whether the location of the baseline PRL ROI corresponded with the location of a T1BH lesion at follow‐up using linearly coregistered images.

The rater and consensus reviewers were blinded to clinical and demographic information. Further information on rater training and reliability analyses are reported elsewhere.^34^ The PRL criteria were consistent with the 2024 NAIMS consensus statement on imaging chronic active lesions, encompassing (1) the presence of a paramagnetic rim contiguous with at least two‐thirds of the outer lesion edge, (2) a diamagnetic core relative to surrounding extra‐lesional white matter, (3) a maximum diameter of ≥3 mm, and (4) non‐enhancement on post‐contrast T1 sequence.^3^ PRLs coincident with confluent T2 lesions were retained if they corresponded with the T2 lesion edge.

Normalized volumes of the brain parenchyma were calculated using SIENAX,^35^ and longitudinal percent brain parenchymal volume changes were calculated using SIENA^17^ with lesion‐filled 3D T1w images.^36^

### Statistical analyses

All statistical analyses were conducted using SPSS version 29.0 (IBM, Armonk, NY, United States). Paired‐sample *t*‐tests were used to test mean longitudinal changes in Expanded Disability Status Scale (EDSS) scores, lesion numbers, and lesion volumes.

#### Model‐based associations between PRLs and T1BH lesions

To test whether PRLs that corresponded with T1BH lesions at baseline were more likely to subsequently disappear, a mixed‐effects model with binary logistic link function was fit with PRL subtype (disappearing or persisting) as the independent variable, T1BH baseline overlap (yes/no) as the dependent variable of interest, a random effect of subject, and covariates of age, sex, and disease duration. Subsequently, the subset of PRLs that did not overlap with baseline T1BH lesions were used to evaluate whether persisting PRLs were more likely than disappearing PRLs to convert to T1BH lesions at follow‐up. A mixed‐effects model with binary logistic link function was fit with T1BH follow‐up overlap (yes/no) as the independent variable, PRL subtype (disappearing or persisting) as the dependent variable of interest, a random effect of subject, and covariates of age, sex, disease duration, and follow‐up time.

#### Model‐based associations between PRLs and demographic, clinical, and radiological characteristics

Linear models were fit using baseline PRL number or change in PRL number as dependent variables, and binary logistic models were fit using per‐subject new PRL appearance (yes/no) as the dependent variable. All models included covariates of baseline age, sex, baseline disease duration, baseline Expanded Disability Status Scale, baseline disease course, baseline T2‐LV, T1‐LV, and baseline Gad‐LV. Models with change in PRL number and new PRL appearance also included follow‐up time and baseline PRL number as covariates. Note that parenchymal brain volume was not included as a covariate in any statistical model. To control for potential confounding age‐related differences in DMT use, we reevaluated associations between baseline PRL number and age after re‐fitting the model on the subset of pwMS on either no baseline DMTs or on low/moderate‐efficacy DMTs (i.e. excluded pwMS on high‐efficacy DMTs), as previously described.^37^

Generalized linear mixed‐effects models, with random effects of “subject,” were fit using per‐PRL change in volume or per‐PRL disappearance (yes/no) as outcome variables. For per‐PRL disappearance models, each PRL that was present at baseline was nominally coded with a value of 0 if it persisted at the overall follow‐up timepoint or a 1 if it disappeared at the follow‐up timepoint. Per‐PRL disappearance models used logistic link functions and were evaluated for fit using pseudo‐*R* ^2^. All models included covariates of follow‐up time, baseline age, sex, baseline disease duration, baseline EDSS, baseline disease course, baseline T2‐LV, baseline Gad‐LV, baseline PRL number, and baseline per‐PRL volume.

#### Model‐based associations between PRLs and DMTs, environmental characteristics, and genotype

For pwMS with available data, baseline DMT efficacy (high vs. none, moderate/low efficacy vs. none, and high vs. moderate/low efficacy), baseline serum ENBA log‐concentration, baseline HLA DRB1*1501 genotype (AA or GA allele vs GG allele), baseline serum calcitriol log‐concentration, baseline total smoking pack‐years, and current smoking status (smoking vs. non‐smoking) were tested for their associations with PRLs. These variables were tested separately (except for pack‐years and smoking status, which were analyzed together) in the models detailed in “Model‐based associations between PRLs and demographic, clinical, and radiological characteristics” with the specified demographic, clinical, and radiological covariates. Model assumptions for all models were assessed using standard diagnostic plots.

#### Power analysis for future clinical trials using PRL disappearance and appearance

G*Power 3.1.9.7 software package was used to estimate sample sizes needed for clinical trials with PRL rim disappearance rate or new PRL appearance as outcome measures.^38^ Sample size calculations were performed using two‐tailed Fisher's Exact Conditional Tests for trial durations of 12, 24, and 36 months, at 80% power and a significance level of 5%. For PRL rim disappearance trials, we performed calculations for the proportion of pooled PRLs disappearing per treatment arm, assuming that PRLs from the same subject can be treated independently as shown previously.^2^ RRMS and progressive MS (PMS) groups were evaluated separately. An inclusion criterion of at least one PRL at baseline was assumed, giving an observed mean of 4.4 baseline PRLs in the RRMS group and 3.6 baseline PRLs in the PMS group. We evaluated sample sizes needed for treatments that increase PRL disappearance rates by 50%, 100%, 200%, and 500%. Pooled proportion rims disappearing were estimated for each trial duration based on our observed annualized rates. An additional analysis was performed based on pwMS on high‐efficacy DMTs at baseline as the control group (RRMS and PMS pooled).

For PRL appearance trials, we evaluated sample sizes needed and for treatments that decrease PRL incidence by 25%, 50%, 75%, and 100%, with 100% corresponding with no new PRL appearance in the treatment group. Control groups of no DMT use or low/moderate efficacy DMT use were considered separately. PwMS of any disease course were included in analyses to maximize the accuracy of PRL appearance rate estimation. New PRL incidence for each trial duration was estimated from our observed annualized new PRL incidence rates (3.85% of pwMS per year in the untreated group, 2.28% of pwMS per year on low/moderate efficacy DMTs).

## Results

### Demographic, clinical, radiological, environmental, and genetic characteristics

The final cohort included 152 pwMS with baseline and follow‐up clinical and MRI data. Baseline values for serum anti‐EBNA‐1 titers were available for 146 pwMS, smoking information for 122 pwMS, *HLA* DRB1*1501 genotyping for 107 pwMS, and serum calcitriol levels for 41 pwMS. Full cohort characteristics are shown in Table 1.

**Table 1:** Demographic and clinical cohort information.

Demographic Information	Baseline	Follow‐up	Change
Number of pwMS	152		–
Sex	111 female, 41 male	111 female, 41 male	–
Age, mean ± SD	45.8 ± 11.7 years	54.0 ± 11.8 years	–
Disease duration, mean ± SD	13.5 ± 10.3 years	21.7 ± 10.4 years	–
Follow‐up time, mean ± SD	–	8.2 ± 2.4 years	–
EDSS, median [IQR]	2.5 [1.5–4.4]	3.5 [2.0–5.5]	***Mean change ± SD*** **0.58 ± 0.97 (*p* < 0.001)** ^**a**^
pwRRMS/pwPMS, *n* (%)	113 (74.3%)/ 39 (25.7%)	103 (67.8%)/ 49 (32.2%)	–
Brain parenchymal volume, mean ± SD	1495.0 ± 97.5 mL	1459.7 ± 101.0 mL	***Annualized % Diff.*** **−0.3% ± 0.4% (*p* < 0.001)** ^**a**^
DMT medication			
*N*	152*	145**	–
High efficacy, *n* (%)	25 (16.4%)	29 (20.0%)	–
Low/moderate efficacy, *n* (%)	108 (71.1%)	96 (66.2%)	–
None, *n* (%)	19 (12.5%)	20 (13.8%)	–
Serum anti‐EBNA‐1 titer			
*N*	146	–	–
Median [IQR]	122.3 [45.3–286.6]	–	–
Smoking			
*N*	122		–
Total pack‐years at baseline, years	***Mean ± SD***: 7.9 ± 13.7 ***Median [IQR]***: 0 [0–11.25]	–	–
Current smoking status, *n* (%)	15 (12.3%)		–
Serum calcitriol concentration			
*N*	41	–	–
Median [IQR]	0.020 ng/mL [0.012–0.033]	–	–
*HLA* DRB1*1501 allele			
*N*	107	–	–
GG, *n* (%)	52 (48.6%)	–	–
GA, *n* (%)	48 (44.9%)	–	–
AA, *n* (%)	7 (6.5%)	–	–

DMT, disease‐modifying therapy; EDSS, Expanded Disability Status Scale; IQR, Interquartile range; pwMS, people with multiple sclerosis; pwPMS, people with progressive multiple sclerosis; pwRRMS, people with relapsing remitting multiple sclerosis; SD, standard deviation. *p* values < 0.05 (in bold) are considered significant.

^a^ Two‐sided paired‐samples *t*‐test.

* At baseline, “Low/Moderate Efficacy” medications included fingolimod (1/152, 0.7%), glatiramer acetate (36/152; 23.7%), interferon beta‐1a (66/152; 43.4%), IVIG immunoglobulin (1/152, 0.7%), mycophenolic acid (2/152, 1.3%). “High Efficacy” medications included azathioprine (1/152, 0.7%), methotrexate (1/152, 0.7%), ocrelizumab (25/152, 16.4%).

** At follow‐up, “Low/Moderate Efficacy” medications included dimethyl fumarate (14/152, 9.2%), fingolimod (5/152, 3.3%), glatiramer acetate (29/152, 19.1%), interferon beta‐1a (32/152, 21.1%), IVIG immunoglobulin (4/152, 2.6%), mycophenolic acid (2/152, 1.3%), peginterferon beta‐1a (4/152, 2.6%), and teriflunomide (5/152, 3.3%). “High Efficacy” medications included methotrexate (1/152, 0.7%), ocrelizumab (23/152, 15.1%), and rituximab (5/152, 3.3%).

Half (50.0%, *n* = 76) of pwMS had at least one PRL at baseline (PRL+). There were 319 total baseline PRLs detected, with a mean of 2.1 ± 3.7 PRLs per pwMS. Of the PRLs present at baseline, 115 (36.1%) disappeared at the follow‐up timepoint. Further information on lesion characteristics are shown in Table 2.

**Table 2:** Lesion characteristics.

	Baseline	Follow‐up	Change
T2‐LV, mean ± SD	10.2 ± 13.4 mL	14.0 ± 16.8 mL	**3.9 ± 6.3 (*p* < 0.001)** ^a^
T1‐LV, mean ± SD	1.7 ± 3.5 mL	2.2 ± 3.8 mL	**0.5 ± 1.3 (*p* < 0.001)** ^a^
Gad LV, mean ± SD	0.07 ± 0.37 mL	0.004 ± 0.03 mL	**−0.04 ± 0.16 (*p* = 0.016)** ^a^
PRL prevalence, % (number of pwMS)	50.0% (76)	44.7% (68)	–
One or more persisting PRLs	–	36.2% (55)	–
One or more disappearing PRLs	–	30.3% (46)	–
One or more newly appearing PRLs	–	18.4% (28)	–
Number of PRLs, mean ± SD (total number of PRLs)	2.1 ± 3.7 (319)	1.7 ± 3.4 (259)	**−0.4 ± 1.9 (*p* = 0.010)** ^a^
Persisting PRLs	–	1.3 ± 2.8 (204)	–
Disappearing PRLs	–	0.8 ± 1.7 (115)	–
Newly appearing PRLs	–	0.4 ± 1.2 (55)	–
Annualized disappearance in PRL+ pwMS at baseline			
Disappearing PRLs per subject, mean ± SD	–	**Overall:** 0.189 ± 0.277 **pwRRMS:** 0.178 ± 0.221 **pwPMS:** 0.229 ± 0.417	–
Proportion total PRLs disappearing, %	–	**Overall:** 4.40% **pwRRMS:** 4.11% **pwPMS:** 5.52%	–
Total PRL volume, mean ± SD	0.6 ± 1.2 mL	0.5 ± 1.2 mL	
Per‐PRL volume, mean ± SD			
Persisting PRLs	0.26 ± 0.22 mL	0.30 ± 0.27 mL	0.02 ± 0.23 mL (*p* = 0.157)^a^
Disappearing PRLs	0.19 ± 0.15 mL	–	–
Newly appearing PRLs	–	0.19 ± 0.17 mL	–

Gad‐LV, gadolinium‐enhancing lesion volume; PRL, paramagnetic rim lesion; pwMS, people with multiple sclerosis; pwPMS, people with progressive multiple sclerosis; pwRRMS, people with relapsing remitting multiple sclerosis; SD, standard deviation; T1‐LV, T1 lesion volume; T2‐LV, T2 lesion volume. *p* values < 0.05 (in bold) are considered significant.

^a^ 2‐sided paired‐samples *t*‐test.

The mean follow‐up time was 8.2 ± 2.4 years. At follow‐up, the mean number of PRLs per pwMS decreased by 0.4 ± 1.9 (p = 0.010). 36.2% (*n* = 55) of pwMS had at least one persisting PRL, 30.3% (*n* = 46) had at least one disappearing PRL, and 18.4% (*n* = 28) had at least one newly appearing PRL.

### Model‐based associations between PRLs and T1BH lesions

Among PRLs present at baseline, 123/319 (38.6%) overlapped with a T1BH lesion at the baseline timepoint, and 106 additional PRLs converted to T1BH lesions at follow‐up (total of 71.8%). PRLs that overlapped with T1BH lesions at baseline were more likely to disappear at follow‐up than PRLs that did not overlap with baseline T1BH lesions (exp(*B*) = 4.451, *p* = 0.041). Among PRLs that did not overlap with T1BH lesions at baseline, persisting PRLs were more likely to convert to T1BH lesions than disappearing PRLs (exp(*B*) = 9.524, *p* = 0.026). All T1BH lesions identified at baseline were also found to be T1BH lesions at follow‐up.

### Model‐based associations between PRLs and demographic, clinical, and radiological characteristics

Table 3 shows the associations between PRLs and demographic, clinical, and radiological pwMS characteristics. Baseline PRL number was associated with younger age (*B* = −0.108 PRLs/year, *p* < 0.001) and greater baseline T2‐LV (*B* = 0.130 PRLs/mL, *p* = 0.010). The association with younger age remained significant when restricting the analysis to pwMS on low/moderate efficacy DMTs or no DMTs at baseline (*B* = −0.066, *p* = 0.010). Odds of new PRL appearance were associated with greater baseline Gad‐LV (exp(*B*) = 49.132 odds per mL, *p* = 0.014). Individual PRLs had greater odds of disappearing with longer follow‐up times (exp(*B*) = odds 1.657 per year, *p* < 0.001) and smaller baseline volume (exp(*B*) = odds 0.003 per mL, *p* < 0.001). The PRL disappearance model without baseline volume or follow‐up time as covariates had pseudo‐*R* ^2^ = 0.050, the model with baseline volume (but without follow‐up time) had pseudo‐*R* ^2^ = 0.186, the model with follow‐up time (but without baseline volume) had pseudo‐*R* ^2^ = 0.140, and the model with all covariates (including follow‐up time and baseline volume) had pseudo‐*R* ^2^ = 0.287. In sensitivity analyses with DMT use added as a nuisance covariate, male sex became significantly associated with greater baseline PRL number (*B* = 1.184 PRLs, *p* = 0.042), and all previously significant associations remained significant.

**Table 3:** Model‐based associations between PRLs and demographic, clinical, and radiological characteristics. exp(*B*) >1 indicates increased odds, exp(*B*) <1 indicates decreased odds, *B* >0 indicates a positive association, and *B* <0 indicates a negative association. Covariates with *p* < 0.05 are bolded.

	Per‐subject			Per‐PRL	
	Baseline PRL number	Change in PRL number	Odds PRL appearance^a^	Odds of PRL disappearance^a^, ^b^	Change in volume^b^
*N*	152 pwMS	152 pwMS	152 pwMS	76 pwMS, 319 baseline PRLs	55 pwMS, 204 persisting PRLs
Follow‐up time	–	*B* = −0.093 PRLs/year *p* = 0.137	exp(*B*) = 0.860 odds/year *p* = 0.131	**exp(*B*) = 1.657 odds/year** ***p* < 0.001**	*B* = −0.016 mL/year *p* = 0.099
Baseline pwMS age	***B* = −0.108 PRLs/year** ***p* < 0.001**	*B* = −0.016 PRLs/year *p* = 0.345	exp(*B*) = 0.971 odds/year *p* = 0.269	exp(*B*) = 1.035 odds/year *p* = 0.258	*B* = −0.003 mL/year *p* = 0.169
Male sex	*B* = 1.175 PRLs *p* = 0.053	*B* = 0.130 PRLs *p* = 0.700	exp(*B*) = 1.481 odds *p* = 0.443	exp(*B*) = 1.529 odds *p* = 0.536	*B* = −0.036 mL *p* = 0.451
Baseline disease duration	*B* = −0.015 PRLs/year *p* = 0.673	*B* = −0.022 PRLs/year *p* = 0.254	exp(*B*) = 1.008 odds/year *p* = 0.806	exp(*B*) = 1.045 odds/year *p* = 0.246	*B* = −0.002 mL/year *p* = 0.539
Baseline EDSS	*B* = 0.063 PRLs/unit increase *p* = 0.750	*B* = −0.018 PRLs/unit increase *p* = 0.875	exp(*B*) = 0.894 odds/unit increase *p* = 0.564	exp(*B*) = 1.584 odds/unit increase *p* = 0.082	*B* = −0.003 mL/unit increase *p* = 0.898
Baseline progressive disease course	*B* = 0.290 PRLs *p* = 0.736	*B* = 0.106 PRLs *p* = 0.824	exp(*B*) = 1.464 odds *p* = 0.671	exp(*B*) = 0.214 odds *p* = 0.144	*B* = 0.068 mL *p* = 0.408
Baseline T2‐LV	***B* = 0.130 PRLs/mL** ***p* = 0.010**	*B* = −0.004 PRLs/ *p* = 0.881	exp(*B*) = 1.040 odds/mL *p* = 0.326	exp(*B*) = 0.986 odds/mL *p* = 0.532	*B* = 0.002 mL/mL *p* = 0.336
Baseline T1‐LV	*B* = −0.015 PRLs/mL *p* = 0.934	*B* = −0.001 PRLs/mL *p* = 0.992	exp(*B*) = 0.970 odds/mL *p* = 0.831	exp(*B*) = 0.890 odds/mL *p* = 0.684	*B* = 0.022 mL/mL *p* = 0.204
Baseline Gad‐LV	*B* = −0.665 PRLs/mL *p* = 0.380	*B* = 0.430 PRLs/mL *p* = 0.304	**exp(*B*) = 49.132 odds/mL** ***p* = 0.014**	exp(*B*) = 1.656 odds/mL *p* = 0.611	*B* = 0.073 mL/mL *p* = 0.411
Baseline total PRL number	–	***B* = −0.219 PRLs/PRL** ***p* < 0.001**	exp(*B*) = 1.073 odds/PRL *p* = 0.228	–	–
Baseline per‐PRL volume	–	–	–	**exp(*B*) = 0.003 odds/mL** ***p* < 0.001**	***B* = −0.283 mL/mL** ***p* < 0.001**

Gad‐LV, gadolinium‐enhancing lesion volume; PRL, paramagnetic rim lesion; pwMS, people with multiple sclerosis; T1‐LV, T1 lesion volume; T2‐LV, T2 lesion volume.

^a^ Model included a logistic link function.

^b^ Model included a random effect of subject.

### Model‐based associations between PRLs and DMTs, environmental characteristics, and genotypes

Table 4 shows the associations between PRLs and DMTs, genetic, and environmental characteristics. 31.6% (6/19) of pwMS not on any DMT at baseline developed at least one new PRL, whereas 19.4% (21/108) on low/moderate‐efficacy DMTs and 12.0% (3/25) of pwMS on high‐efficacy DMTs developed a new PRL. In corrected models, high‐efficacy DMT use at baseline (exp(*B*) = 0.088 odds, *p* = 0.024) and low/moderate‐efficacy DMT use at baseline (exp(*B*) = 0.269 odds, *p* = 0.049) were associated with reduced odds of new PRL appearance compared to no DMT use at baseline. When restricting analyses to pwMS who did not change DMT efficacy from baseline to follow‐up (n = 96), high‐efficacy use was still associated with reduced odds of PRL appearance compared to no DMT use (exp(*B*) < 0.001 odds, *p* < 0.001).

**Table 4:** Model‐based associations between PRLs and DMTs, environmental characteristics, and genotype. exp(*B*) >1 indicates increased odds, exp(*B*) <1 indicates decreased odds, *B* >0 indicates a positive association, and *B* <0 indicates a negative association. Covariates with *p* < 0.05 are bolded.

	Per‐subject			Per‐PRL	
	Baseline PRL number	Change in PRL number	Odds PRL appearance^a^	Odds of PRL disappearance^a^, ^b^	Change in volume^b^
Baseline DMT efficacy					
High vs. none	*B* = 1.582 PRLs *p* = 0.129	*B* = −0.319 PRLs *p* = 0.571	**exp(*B*) = 0.088 odds** ***p* = 0.024**	exp(*B*) = 0.896 odds *p* = 0.933	*B* = −0.028 mL *p* = 0.766
Low/moderate vs. none	*B* = −0.122 PRLs *p* = 0.883	*B* = −0.030 PRLs *p* = 0.946	**exp(*B*) = 0.269 odds** ***p* = 0.049**	exp(*B*) = 1.077 odds *p* = 0.947	*B* = −0.013 mL *p* = 0.878
HLA DRB1*1501 allele					
AA vs. GG	*B* = −0.760 PRLs *p* = 0.573	*B* = 0.784 PRLs *p* = 0.202	exp(*B*) = 0.687 odds *p* = 0.771	exp(*B*) <0.001 odds *p* = 0.982	*B* = 0.198 mL *p* = 0.073
GA vs. GG	*B* = 0.124 PRLs *p* = 0.851	*B* = 0.327 PRLs *p* = 0.275	exp(*B*) = 1.557 odds *p* = 0.479	exp(*B*) = 1.730 odds *p* = 0.489	*B* = 0.050 mL *p* = 0.362
Baseline anti‐EBNA‐1 titer	*B* = 4.4E‐4 PRLs/units/mL *p* = 0.702	*B* = 0.063 PRLs/units/mL *p* = 0.584	exp(*B*) = 1.110 odds/unit *p* = 0.636	exp(*B*) = 0.792 odds/units/mL *p* = 0.473	*B* = 0.010 mL/units/mL *p* = 0.633
Smoking^c^					
Baseline pack‐years	*B* = −0.006 PRLs/pack‐year *p* = 0.744	*B* = −0.003 PRLs/pack‐year *p* = 0.803	exp(*B*) = 0.984 odds/pack‐year *p* = 0.547	exp(*B*) = 1.000 odds/pack‐year *p* = 0.994	*B* <0.001 mL/pack‐year *p* = 0.948
Current smoking status (smoker vs. non‐smoker)	***B* = 2.294 PRLs** ***p* = 0.005**	*B* = −0.376 PRLs *p* = 0.429	exp(*B*) = 0.046 odds *p* = 0.066	exp(*B*) = 0.317 odds *p* = 0.347	*B* = −0.028 mL *p* = 0.747
Baseline serum calcitriol concentration	*B* = 12.133 PRLs/ng/mL *p* = 0.767	*B* = −11.640 PRLs/ng/mL *p* = 0.342	exp(*B*) <0.001 odds/ng/mL *p* = 0.406	exp(*B*) >1E5 odds/ng/mL *p* = 0.270	*B* = −29.821 mL/ng/mL *p* = 0.173

DMT, disease‐modifying therapy; EBNA, Epstein–Barr nuclear antigen 1; PRL, paramagnetic rim lesion.

^a^ Model included a logistic link function.

^b^ Model included a random effect of subject.

^c^ “Smoking” models included baseline pack‐years and current smoking status.

Current smoking status was associated with higher baseline PRL number (*B* = 2.294, *p* = 0.005), but baseline pack‐years was not (*B* = −0.006, *p* = 0.744). *HLA* DRB1*1501 genotype, baseline serum anti‐EBNA‐1 titer concentration, and baseline calcitriol serum concentration were not associated with baseline PRLs or PRL evolution (*p* > 0.05).

### Power analysis for PRL rim disappearance and appearance

Table 5 shows sample size estimates for various levels of treatment effects and various trial durations. For PRL disappearance rate trials, estimated per‐group sample sizes ranged from 2 pwPMS in a 36‐month trial of a drug with 500% increase in efficacy to 430 pwRRMS in a 12‐month trial of a drug with 50% increase in efficacy. For PRL appearance incidence trials, our smallest estimated sample size was 67 pwMS per group for a 36‐month trial and 100% decrease in PRL appearance (i.e., no new PRLs in the treatment group), compared to a control group not on any DMTs. Table S1 shows the sample size estimates for PRL disappearance trials with pwMS on high‐efficacy DMTs at baseline as the control group (RRMS and PMS pooled).

**Table 5:** Sample size estimations for clinical trials using PRL rim disappearance as an outcome measure. Each estimation was performed at a power of 80% and a significance level of 5%.

PRL disappearance				PRL appearance			
Disease group	Duration	Treatment effect (%)	Number of pwMS per group	DMT group^a^	Duration	Treatment effect (%)	Number of pwMS per group
RRMS	12 months	50	430	None	12 months	25	5721
		100	129			50	1259
		200	43			75	462
		500	12			100^b^	204
	24 months	50	204		24 months	25	2752
		100	62			50	609
		200	20			75	229
		500	5			100^b^	102
	36 months	50	129		36 months	25	1771
		100	38			50	395
		200	13			75	151
		500	3			100^b^	67
PMS	12 months	50	330	Low/moderate efficacy	12 months	25	9794
		100	103			50	2153
		200	34			75	808
		500	9			100^b^	346
	24 months	50	156		24 months	25	4781
		100	47			50	1054
		200	15			75	389
		500	4			100^b^	172
	36 months	50	95		36 months	25	3125
		100	28			50	696
		200	9			75	259
		500	2			100^b^	115

pMS, people with multiple sclerosis; PMS, progressive multiple sclerosis; PRL, paramagnetic rim lesion; RRMS, relapsing–remitting multiple sclerosis.

^a^ All disease courses (RRMS and PMS) included in analyses.

^b^ Corresponds with no new PRLs observed over the trial duration.

## Discussion

In this longitudinal study of 152 pwMS, we evaluated clinical, radiological, environmental, and genetic factors associated with PRL evolution and identified DMT use as effective in reducing new PRL incidence, but not rim disappearance. We also described the 8‐year rates of new PRL appearance and rim disappearance and calculated sample sizes necessary for future clinical trials targeting these markers. These findings are an important step in understanding the biological underpinnings of chronic active inflammation and for clinical translation of PRLs as an imaging biomarker.

We found that baseline PRLs persisted at follow‐up were more likely to convert to T1BH lesions than PRLs that disappeared. This indicates that PRL disappearance may be associated with preservation of underlying WM tissue integrity, potentially from cessation of local chronic inflammation. Additionally, we found that PRLs that overlapped with T1BH lesions at baseline were more likely to disappear at follow‐up than PRLs that did not overlap with baseline T1BH lesions. It is unclear why this is the case, but may be because T1BH‐overlapping PRLs represent an older subset of PRLs.

Post‐mortem pathological studies have showed that CALs are present in higher numbers in people with long disease durations and in pwPMS compared to pwRRMS.^38^ In contrast, we found that PRLs were more numerous in pwMS of younger age, and did not see an association between PRLs (including PRL appearance and disappearance) and disease course. The reason for this discrepancy is unclear, although it may be because PRLs represent only a subset of histologically defined CALs and may therefore have a different population distribution. The association between PRL number and younger age remained significant in the subset of pwMS on no baseline DMTs or low/moderate‐efficacy DMTs, indicating that greater high‐efficacy DMT use in older pwMS did not explain these results. Additionally, we found that 36% of PRL rims present at baseline disappeared over the mean follow‐up time of 8.2 years, which is lower than a 7‐year median PRL survival time reported in a small study of 70 PRLs in 10 pwMS.^2^ The mechanism by which the microglia in PRL rims dissipate has not been elucidated and it is possible that the iron rim sign persists in a subset of resolved CALs. Histological evaluation of a large sample of PRLs could confirm or deny this possibility. If true, novel imaging methods that distinguish active from inactive PRLs are needed to increase the sensitivity of PRLs as a prognostic marker.

Higher baseline Gad‐LV predicted greater odds of new PRL appearance, consistent with the known pathway from acute contrast‐enhancing lesions to PRLs.^11^, ^12^ Furthermore, we found that baseline DMT use was associated with decreased odds of new PRL appearance. We did not see a significant difference between low/moderate efficacy DMTs (primarily glatiramer acetate and interferon beta‐1a) and high‐efficacy DMTs (primarily ocrelizumab) in decreased PRL appearance, although high‐efficacy DMTs were numerically associated with a more pronounced decrease. Given these data, use of currently available DMTs may be the best available tool to prevent PRL formation, which may be mediated by a reduction in acute lesions. Therefore, early MS diagnosis and DMT initiation may be crucial in preventing chronic compartmentalized disease activity. In contrast, we found that DMT use was not associated with PRL rim disappearance or reducing PRL size. This highlights the need for novel DMTs that target existing compartmentalized brain inflammation. Note that our results are observational and need to be confirmed in future interventional studies.

Current smoking status, but not total pack‐years, was associated with greater number of PRLs at baseline. Smoking is associated with increased early MS disease activity and faster transition to progressive MS.^1^, ^16^ Together, these findings support that greater ongoing acute inflammatory activity leads to greater chronic active inflammation. We did not obtain evidence for associations between PRLs and other factors associated with greater susceptibility to MS, for example, baseline serum anti‐EBNA‐1 or calcitriol levels, or HLA DRB1*1501 genotype. This leaves open the question of which additional factors are associated with chronic active inflammation.

Our power analyses estimated that 12 pwRRMS or 9 pwPMS per group are required to detect a 500% relative increase in PRL disappearance rate in a 12‐month trial. This is comparable to a previous estimate of 16 pwMS per group for a similar treatment effect (i.e., disappearance proportion from 0.02 to 0.12, a 500% relative increase) and trial duration.^2^ However, a drug with only a 50% increase in PRL disappearance rate would require 430 pwRRMS or 330 pwPMS for a 12‐month trial. In comparison, a previous study in pwSPMS estimated that only 57 pwMS would be needed for a trial of the same treatment effect, duration, and power using brain atrophy (measured with SIENA). PRL appearance rate trials required even more participants. The optimal case (i.e., 100% decrease in PRL appearance and no DMT use in the comparator group) was estimated to require 67 pwMS per group for a 36‐month trial. In sum, rim disappearance and appearance may be impractical markers for DMTs with only moderate efficacy in reducing or preventing chronic active inflammation, particularly in phase II trials. Caution should be exercised when considering rim appearance and disappearance as outcome measures in future trial designs.

When planning future clinical trials, several parameters may be chosen to potentially reduce required sample sizes. For example, eligibility criteria could be restricted to pwMS with the presence of PRL, more aggressive disease and/or increased history of inflammatory activity. However, this would have the effect of reducing the generalizability of the findings. Additionally, use of other PRL features as endpoints, such rim susceptibility values, may enable more sensitive evaluation of DMT effects (compared to appearance or disappearance of PRL rims) and should be explored in future studies. Finally, it should be noted that, for the sake of increased statistical efficiency, future clinical trials should employ stratified analysis and implement stratified randomization.

Further standardization of PRL classification may improve PRL classification accuracy and reduce sample sizes needed for clinical trials. Recently released guidelines provide an outline for classification guidelines,^3^ but PRL detection still varies widely between groups, with different groups using different MR image types for PRL detection (e.g., QSM, phase, and susceptibility weighted‐imaging).^34^ Further advances in automated PRL detection, such as with deep learning approaches (possibly combined with synthetic MRI approaches and post‐processing methods such as chi‐separation),^39^, ^40^ may improve between‐center precision. Additionally, PRLs could be combined with other prognostic MRI markers, such as SELs,^41^ and blood‐based biomarkers, such as neurofilament light or glial acidic protein.^42^, ^43^ A major limitation of this study the lack of serial MRI at regular intervals. This prevented detailed analysis of PRL volume trajectories and likely led to underestimation of PRL appearance rates. The lack of pre‐baseline scan limited our ability to assess whether PRLs were relatively new lesions, which can resolve within 4 months of appearance,^5^ or chronically active lesions. Additionally, our study used 3T MRI, which is less sensitive in detecting PRLs than 7T MRI. However, 7T MRI is not widely clinically available so are results are likely directly applicable for large, multicenter clinical trials. Ongoing innovations in MRI acquisition, such as synthetic MRI approaches—which generate contrast‐weighted images based on tissue property measurements—and automated image detection,^44^, ^45^ may further enhance the sensitivity of detecting PRLs at 3T and even 1.5T field strengths. These advancements could also potentially accelerate the clinical translation of PRLs. Another limitation of our study is the small number of pwMS with available serum calcitriol levels, and only seven pwMS possessed the high‐risk AA allele for HLA‐DRAB1*1501. Furthermore, we did not have sufficient information on pre‐baseline DMT start dates to assess the duration of DMT use at baseline. Future studies with larger numbers of pwMS with these data and characteristics may therefore be warranted. Additionally, we did not have access to subject genotypes of risk alleles for MS progression severity, such as rs10191329 in the *DYSF–ZNF638* locus.^14^ Finally, our study was observational, so conclusions about impact of modifiable factors (e.g., DMT use) should be interpreted with caution.

## Conclusions

DMT use is associated with lower odds of new PRL appearance in MS, but not rim disappearance. This highlights the importance of early MS diagnosis and medication initiation, and the need for novel DMTs targeting existing chronic active inflammation. However, caution should be exercised when using PRL rim disappearance as an outcome measure in phase II clinical trials, because cohort sizes become prohibitively large for moderate efficacy investigational drugs.

## Funding Information

Research reported in this publication was supported by grants from the National Institutes of Health (R01NS114227 from the National Institute of Neurological Disorders and Stroke and UL1TR001412 from the National Center for Advancing Translational Sciences). The content is solely the responsibility of the authors and does not necessarily represent the official views of the National Institutes of Health.

## Conflict of Interest

Nothing to report.

## Author Contributions

J.A.R., N.B., and R.Z. contributed to the conception and design of the study. J.A.R., A.B., D.J., M.M., N.B., F.Sc., G.E.W., M.R., S.E., D.H., M.G.D., and R.Z. contributed to the acquisition and analysis of data. J.A.R., N.B., F.Sc., B.W.G., F.B., M.G.D., and R.Z. contributed to drafting the text and preparing the figures.

## Supporting information


Table S1.

## Data Availability

The data that support the findings of this study may be available on reasonable request.
