# Supplementary material for: Determinants of long‐term paramagnetic rim lesion evolution in people with multiple sclerosis
Source: Ann Clin Transl Neurol. 2024 Nov 18;12(2):267–79. doi: 10.1002/acn3.52253 (PMC11822801; doi:10.1002/acn3.52253)
Supplement: Supplementary file 1 — Table S1. [file ACN3-12-267-s001.docx]

**Supplemental Table 1.** Sample size estimations for clinical trials using PRL rim disappearance as an outcome measure with a control group of pwMS taking high-efficacy DMT. Each estimation was performed at a power of 80% and a significance level of 5%, and included RRMS and PMS in a pooled analysis.

| **PRL disappearance** | | |
| --- | --- | --- |
| **Duration** | **Treatment effect** | **Number of pwMS per group** |
| 12 months | 50% | 1124 |
|  | 100% | 337 |
|  | 200% | 110 |
|  | 500% | 28 |
| 24 months | 50% | 513 |
|  | 100% | 152 |
|  | 200% | 48 |
|  | 500% | 11 |
| 36 months | 50% | 310 |
|  | 100% | 90 |
|  | 200% | 26 |
|  | 500% | 7 |

**Legend:** PRL – paramagnetic rim lesion, pwMS – people with multiple sclerosis, PMS –progressive multiple sclerosis, RRMS –relapsing remitting multiple sclerosis.
